# A positive readout single transcript reporter for site-specific mRNA cleavage

**DOI:** 10.7717/peerj.3602

**Published:** 2017-07-20

**Authors:** Nikolay Kandul, Ming Guo, Bruce A. Hay

**Affiliations:** 1 Division of Biology and Biological Engineering, California Institute of Technology, Pasadena, CA, United States of America; 2 Departments of Neurology and Molecular and Medical Pharmacology, UCLA David Geffen School of Medicine, University of California, Los Angeles, CA, United States of America

**Keywords:** Poly(A), Translation, Positive-readout, RNA hairpins, CopT-CopA, Auto-repression, Reporter

## Abstract

Cleavage of mRNA molecules causes their rapid degradation, thereby playing an important role in regulation of gene expression and host genome defense from viruses and transposons in bacterial and eukaryotic cells. Current negative-readout, and repressor-based positive-readout reporters of mRNA degradation have limitations. Here we report the development of a single transcript that acts as a positive reporter of mRNA cleavage. We show that placement of bacterial CopT and CopA hairpins into the 5′ UTR and 3′ UTR of an mRNA results in inhibition of translation of the intervening coding sequence in *Drosophila*. An internal poly(A) tract inserted downstream of the coding sequence stabilizes transcripts cut within the 3′ UTR. When these components are combined in a transcript in which targets sites for RNA cleavage are placed between the poly(A) tract and CopA, cleavage results in translational activation, providing a single transcript-based method of sensing mRNA cleavage with a positive readout.

## Introduction

Cleavage of messenger RNA (mRNA) results in its rapid degradation, regulating turnover and abundance of both prokaryotic and eukaryotic mRNAs. In bacteria, short antisense RNA molecules guide specific cleavage and degradation of complementary RNAs by forming double-stranded RNA species that are recognized and cut by RNase III (Nicholson, 2014). Bacterial antisense RNA molecules act as anti-toxins in some toxin-antitoxin pairs, regulate bacterial immunity, communication, and plasmid propagation (Gerdes & Wagner, 2007). In eukaryotes, microRNAs (miRNAs) loaded into an RNA-induced silencing complex (RISC) guide specific cleavage and degradation of complementary endogenous mRNAs by Argonaute 2 (Ago2) protein in RISC (Ipsaro & Joshua-Tor, 2015). Other classes of small eukaryotic RNA molecules, including piwi-interacting RNA (piRNA), repeat associate small interfering RNA (rasiRNA), and endogenous small interfering RNA (en-siRNA), also cleave host mRNAs in a sequence-guided manner (Goh et al., 2015; Zhang et al., 2015), thereby maintaining adaptive immunity of the eukaryotic cell against active transposons and viral infection (Han et al., 2015; Malone & Hannon, 2009). Sequence specific RNA cleavage also plays diverse roles in gene expression and regulation, host defense and communication, and genome surveillance in bacterial and eukaryotic cells (Fozo, Hemm & Storz, 2008; Gerdes & Wagner, 2007; Nicholson, 2014).

Study of RNA cleavage would benefit from the creation of tools to monitor specific RNA cleavage events *in vivo*. Traditional negative-readout reporters of mRNA cleavage and degradation consist of a constitutively expressed reporter (*eGFP* (Brennecke et al., 2003; Palliser et al., 2006), *lacZ* (Mansfield et al., 2004), and *Gluc* (Kim et al., 2009)) bearing sequences complementary to a specific miRNA or small interfering RNA (siRNA) in the 3′ UTR. The presence of the miRNA or siRNA reduces expression of the transgene reporter by promoting degradation of reporter transcripts (Brennecke et al., 2003; Kim et al., 2009; Mansfield et al., 2004; Palliser et al., 2006) via cleavage (Goh et al., 2015; Zhang et al., 2015), deadenylation (Behm-Ansmant et al., 2006; Piao et al., 2010; Wu, Fan & Belasco, 2006) and decapping (Ameres & Zamore, 2013; Behm-Ansmant et al., 2006). Because negative reporters require degradation of the reporter protein in locations where the reported miRNA/siRNA is expressed, while the reporter protein is synthesized at high levels in other locations, they may be slow to respond.

Available positive-readout systems for reporting siRNA (Lin et al., 2011; Liu et al., 2009) and miRNA levels (Xie et al., 2011) utilize two components, and work by promoting degradation of a transcript that encodes a repressor of reporter expression. Repressor-based positive-readout systems also have several limitations. First, off-target effects in cells may be triggered by expression of heterologous repressors that bind the tetracycline responsive element (Lin et al., 2011; Xie et al., 2011) or the *lac* repressor (Liu et al., 2009; Stevenson et al., 2013). Second, expression of two foreign proteins *in vivo* may also result in an unwanted immune response. Third, because a reporter’s activation depends on repressor mRNA degradation, the repressor protein must be constitutively expressed and have a short half-life. Finally, the efficiency of a repressor-based system depends on an optimal stoichiometric ratio between expression levels of the repressor and the reporter (e.g., Lakshmi & Rao, 2009; Liu et al., 2009; Ryu, Olson & Arnosti, 2001).

We sought to develop a positive reporter of specific mRNA cleavage that relies only on RNA components embedded within a single transcript. Our general strategy was to create a transcript in which translational repression of the reporter mediated by RNA secondary structure formation could be relieved or prevented from forming through cleavage at a specific site within the mRNA molecule.

## Material and Methods

### Assembling of FLuc constructs

**Figure 1:**
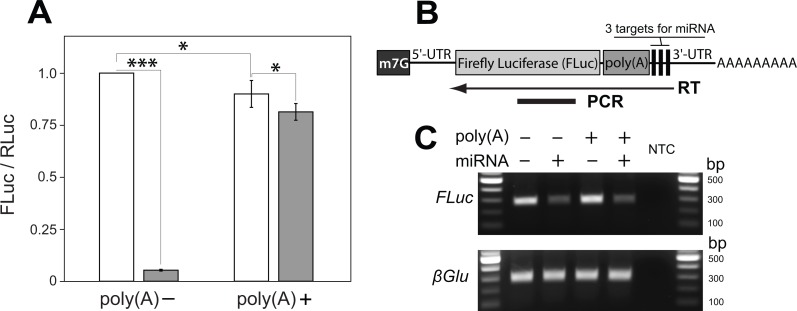
A poly(A) tract placed upstream of miRNA cleavage sites stabilizes the cut transcript. (A) An artificial micro RNA (miRNA) guided cleavage at three complementary sites inserted into the 3′ UTR of Firefly Luciferase (FLuc) transcripts. *Renila* Luciferase (RLuc) served as a reference control and was expressed from a separate plasmid. The FLuc/RLuc ratio was quantified for two kinds of *FLuc* transcripts: without and with an internal poly(A) tract of 139 nucleotides inserted upstream of the miRNA targets sites (poly(A)− and poly(A)+, respectively). The miRNA was either absent (white bars) or present (grey bars) in transfected cells. Bars depict mean ± one standard deviation (*P* < 0.05^∗^ and *P* < 0.001^∗∗∗^). (B–C) RT-PCR was used to analyze the miRNA-guided cleavage of *FLuc* transcripts. (B) *FLuc* transcript is depicted with a 7-methylguanosine cap (m7G) and a poly(A) tract internally and at its 3′ end, respectively. To analyze cleavage of *FLuc* transcripts, a specific primer was used for reverse transcriptase (RT) reaction. The arrow indicates a position and direction of first-strand cDNA synthesis relatively to *FLuc* PCR amplicon and transcript parts. *FLuc* RT-PCR amplicons will not amplify from transcripts cut at miRNA target sites. (C) Co-expression of the miRNA substantially decreased *FLuc* RT-PCR amplification from both kinds of *FLuc* transcripts, and did not affect levels of polyadenylated *β glucuronidase* (*βGlu*) transcripts. No template was added to the last well (NTC).

**Figure 2:**
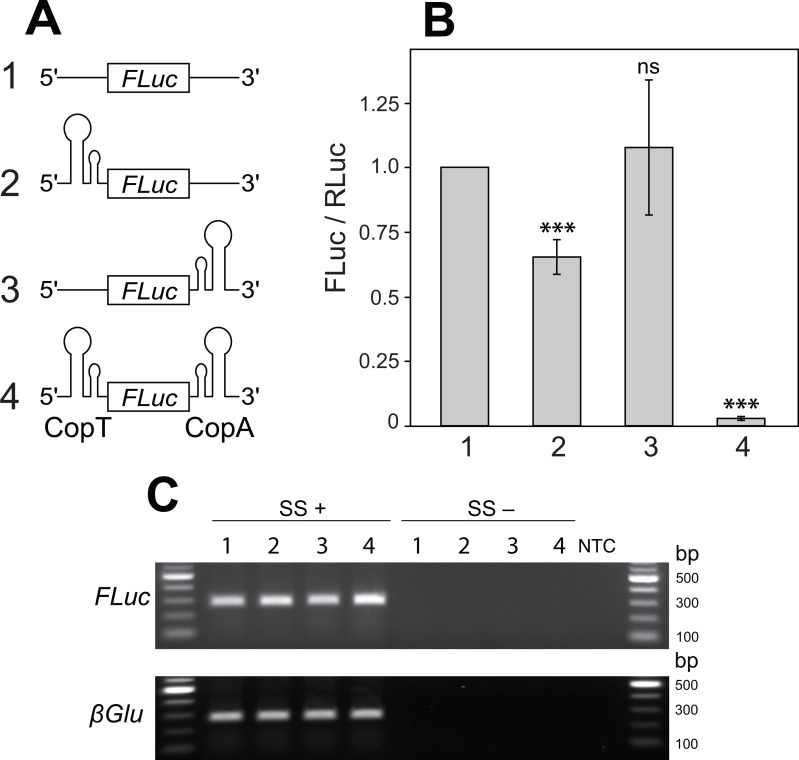
Two mRNA complementary hairpin structures (CopT and CopA) flanking a coding sequence block its translation. (A) No hairpin (1), one (2 and 3) or both hairpin structures (4) were inserted into the 5′ and 3′ UTRs of *FLuc* transcripts, as illustrated. (B) CopT hairpin inserted in the 5′ UTRs significantly decreased FLuc expression (2). Addition of CopA into the 3′ UTRs caused a further drop in the amount of FLuc (4), whereas a single CopA in the 3′ UTRs had no effect (3). Bars depict mean ± one standard deviation (*P* > 0.05 (ns), *p* < 0.01^∗∗^, *P* < 0.001^∗∗∗^). (C) Insertion of one or both hairpin structures did not affect abundances of *FLuc* and *βGlu* transcripts. SuperScript™ III reverse transcriptase was either added (SS+) or not added (SS−) to RT reactions. No template was added to the last well (NTC).

**Figure 3:**
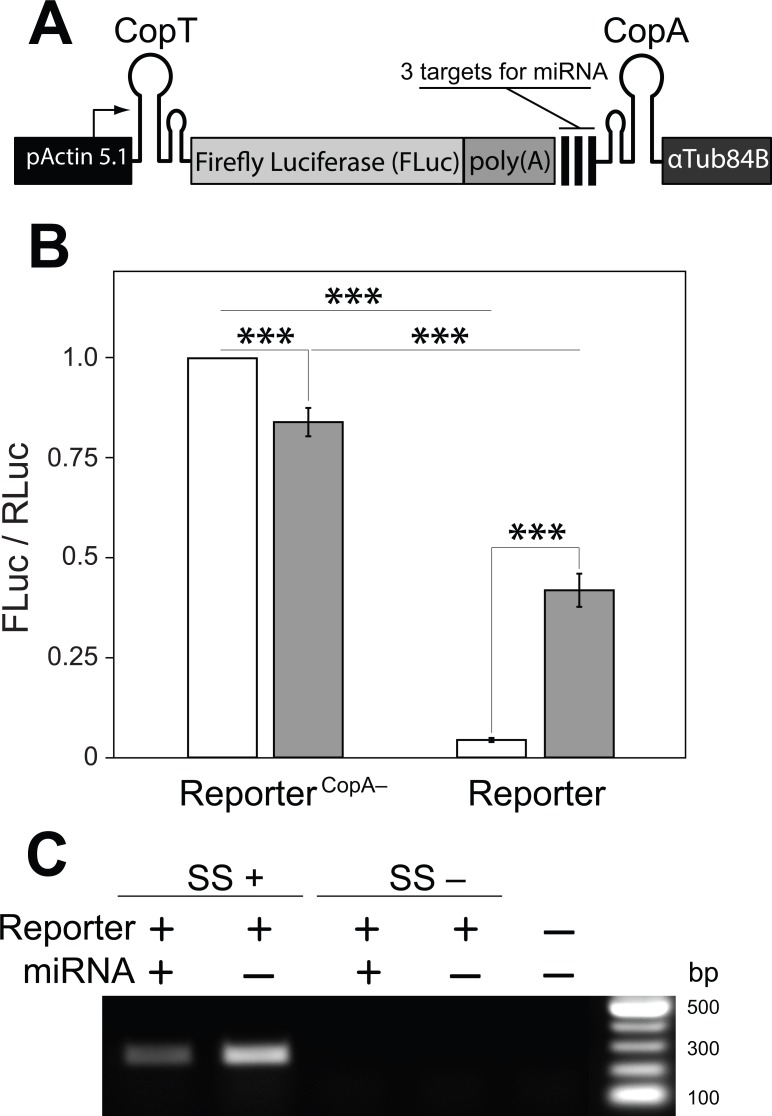
A single transcript reporter of mRNA cleavage. (A) A schematic map of the reporter construct. pActin 5.1 promoter directs constitutive transcription of *FLuc* mRNA. (B) The reporter’s operation was quantified as compared with a non-functional reporter^CopA−^ in the absence (white) and presence (grey) of the miRNA. Addition of the miRNA resulted in a ten-fold increase in FLuc expression from the miRNA reporter. reporter^CopA−^ transcripts express less FLuc in presence of the miRNA. Bars depict mean ± one standard deviation (*P* < 0.01^∗∗^ and *P* < 0.001^∗∗∗^). (C) RT-PCR was used to analyze the abundance of the full-length reporter transcript (see Fig. 1B). Co-expression of the miRNA with the reporter resulted in a substantial decrease in the amount of *FLuc* RT-PCR product. SuperScript™ III reverse transcriptase was either added (SS+) or not added (SS−) to RT reactions. No template was added to the last well (NTC).

A one-step assembling protocol (Gibson et al., 2009) was used to clone parts of constructs and insert unique restriction sites between each functional element. The actin 5.1 promoter from pAc5.1/V5-HisB (Invitrogen, Carlsbad, CA, USA) and firefly (*Photinus pyralis*) luciferase (FLuc) from pGL3 (Promega, Madison, WI, USA) were amplified and cloned into the pBS-C5 vector (GenBank: EF090402). The 3′ UTR of αTubulin 84D (FlyBase, CG2512) was amplified from genomic DNA of *Drosophila melanogaster* using primers 5′-GCGTCACGCCACTTCAACGCTCG-3′ and 5′-AAAGAAAAACAGTGGGGTTTTCTT ATTTCTGAC-3′, and inserted 3′ to the luciferase coding unit. A fragment with three targets perfectly complementary to the guide strand of the artificial miRNA was built from ultramer oligos synthesized by Integrated DNA Technology (IDT^®^) and then cloned upstream of the 3′ UTR (Fig. 1B). We built a fragment with the poly(A) tract in the middle using a two-step PCR amplification of a 200 bp-long IDT^®^ ultramer oligo, which carried 139 bases of Thymine surrounded by sequences complementary to a destination vector, and two short forward and reverse oligos complementary to the unique end sequences of the ultramer oligo (Table 1). This fragment was then cloned between *FLuc*’s coding sequence and the miRNA target sequences (Fig. 1B). CopT and CopA sequences (Kolb et al., 2000b) were built from ultramer oligos synthesized by IDT^®^. To test whether inclusion of one or both hairpin structures into the FLuc transcript affects the expression of FLuc, CopT and/or CopA were cloned into the *FLuc* construct without the miRNA targets and the internal poly(A) tract (Fig. 2). To build the miRNA reporter (GenBank: KY412813), a fragment bearing the miRNA target sites and the poly(A) tract was cloned between the *FLuc* coding sequence and CopA in the *FLuc* construct carrying both CopT and CopA (Fig. 3A). To build the control reporter (reporter^CopA−^), CopA was replaced with a random sequence of an equal size (GCCACTGATATGACCAGTACAACACGTATCCGTGATACGTTACGCAGGATATTAAATATACCTCTAACAACGGCATTGGAGTATAAGTCT).

**Table 1:** Primers used to build and clone the poly(A) tract.

Primer name	Length (bp)	Sequence
Reverse long oligo	200	5′-ATTGTTCGAACTCGAGGAGTGGCGGCCGCTTTTTTTTTTTTTTTTTTTTTTTTTTTTTTTTTTTT TTTTTTTTTTTTTTTTTTTTTTTTTTTTTTTTTTTTTTTTTTTTTTTTTTTTTTTTTTTTTTTTTTTTT TTTTTTTTTTTTTTTTTTTTTTTTTTTTTTTTTTGGATCCGCCTCGAATTCTTACACGGCGATCTT-3′
Forward short oligo	32	5′-AAGATCGCCGTGTAAGAATTCGAGGCGGATCC-3′
Reverse short oligo	27	5′-ATTGTTCGAACTCGAGGAGTGGCGGCC-3′

**Notes.**

The table lists three primer sequences that were used to build the poly(A) tract.

### Assembling of RLuc construct

To build a control reporter construct, the coding sequence of *Renilla reniformis* luciferase (RLuc) was amplified from pRL (Promega, Madison, WI, USA) and cloned between the pAc5.1 promoter and the 3′ UTR of αTubulin 84D (GenBank: KY412814).

### Assembling of miRNA construct

A fragment with a coding sequence for the far-red fluorescent protein mKate2 and a downstream synthetic intron, which includes within it a single miRNA precursor, was synthesized by DNA2.0 and cloned into the pAc5.1/V5 HisB vector between *EcoRI* and *NotI* sites to build the miRNA construct (GenBank: KY412815). The miRNA was designed following the miR-6.1 method (Chen et al., 2007) to encode a mature guide miRNA of the following sequence: ATTGTTCGAACTCGAGGAGTGG. No off-target sites in the *Drosophila* genome (FB2016_03) were identified as determined using E-RNAi (Horn & Boutros, 2010) and GESS (Yilmazel et al., 2014) programs, or by nucleotide BLAST search of the guide sequence.

### Cell culture maintenance and transfection

*Drosophila* S2 cells were maintained using standard protocols (Invitrogen #R690-07). Prior to transient transfection, cells were seeded in a 24-well plate at the density of 300,000–400,000 cells per well and grown for 12 h. A transfection complex contained 800 ng of total DNA and 2.0 μl of FuGene^®^ HF (Promega, Madison, WI, USA) in 50 μl of DEPC treated water. The complex was incubated for 30 mins at room temperature before applying it to S2 cells. To enable comparison between different experiments, the same amounts of *FLuc*, *RLuc*, and miRNA-expressing constructs were used in all experiments: 400 ng, 50 ng, and 100 ng, respectively. One half of experimental wells received the miRNA construct while the other half did not. An empty *pHsp70* vector (*pCaSpeR-hs*) provided the remaining 250 ng of transfected DNA, or 350 ng when the miRNA construct was not added. Two wells (technical replicates) were transfected with 24 μL of the same transfection complex. All transfection experiments were repeated at least four times.

### Luciferase reporter assay and data analysis

The dual-Luciferase reporter (DLR) assay by Promega was used for quantification of FLuc and RLuc activities. The cells were collected from a plate well into an Eppendorf tube 30 h post-transfection, pelleted at 830 rcf for 1 min, washed with PBS buffer, and pelleted again. The pelleted cells were resuspended in 115 μl of a freshly made passive lysis buffer (DLR, Promega, Madison, WI, USA), lysed for 20 mins while shaking, and frozen at −80°C. Standard protocols were then used to determine activities of both luciferases using the GloMax 20/20 luminometer (Promega, Madison, WI, USA). FLuc/RLuc relative activities were normalized to that of a control and averaged for two technical replicates. An average ± one-standard deviation was calculated for normalized fold differences from at least four separate transfections.

### RT-PCR analysis of miRNA-guided cleavage of *FLuc* transcripts

RT-PCR with a specific reverse transcriptase (RT) primer complementary to a sequence 3′ of the three miRNA target sites was used to analyze the abundance of uncut (full-length) *Fluc* transcripts (Fig. 1B). Total RNA was extracted from S2 cells 30 h post-transfection using the mirVana™ miRNA isolation Kit (Ambion, Foster City, CA, USA). To remove DNA contamination, 3 μg of total RNA was treated with the Turbo DNA-free™ kit (Ambion). Then cDNA was synthesized from 500 ng of RNA using SuperScript™ III (SSIII) reverse transcriptase (Invitrogen, Waltham, MA, USA) and the RT primer, 5′-CTCGAGGAGTGGGACTGATACG-3′. Equal amounts of RT products were used to PCR amplify a 277 bp-fragment of *FLuc* coding sequence using the following primers: 5′-GAAGCGAAGGTTGTGGATCTGG-3′ and 5′-GGTGTTGGAGCAAGATGGATTCC-3′ with 27 amplification cycles (Fig. 1B). The amount of *FLuc* PCR product is expected to be proportional to the amount of uncut transcript because the PCR amplified 277 bp-fragment of *FLuc* is located upstream from the miRNA target sites (Fig. 1B). ImageJ 2.0 was used to estimate a percentage difference in amounts of PCR products from a gel image. We measured and compared average pixel brightnesses for identical areas, which corresponded to a PCR band, in different gel lanes.

To assess whether miRNA expression affects the overall abundance of polyadenylated transcripts in *Drosophila* S2 cells, a 278 bp-fragment of *β glucuronidase* (*βGlu*) transcript was amplified by PCR from 2 μL of oligo(dT) _20_-synthesized cDNA using the following primers: 5′-AATAAGGATAGTGAGAGGTGCGATATG-3′ and 5′-CCTGTATAATCTTCAATTCGAGCTGTT-3′, with 30 amplification cycles.

### RT-PCR analysis of transcript stability

To test whether addition of one or both hairpin RNA sequences (CopA and CopT) caused degradation of FLuc transcripts, we analyzed relative abundance of different FLuc transcripts (Fig. 2A) using RT-PCR. The same protocol as above was used for the total RNA extraction and the removal of potential DNA contamination. Then cDNA was synthesized from 500 ng of RNA using SSIII reverse transcriptase (Invitrogen, Waltham, MA, USA) and an oligo(dT)_20_ primer. To control for DNA contamination, a replicate of each experimental RT reaction was also run without SS III reverse transcriptase (SS- control). Equal amounts of RT products were used to PCR amplify the *FLuc* fragment with 25 amplification cycles.

### Statistical methods

Statistical analysis was performed in JMP^®^ 8 (SAS Institute Inc, Cary, NC, USA). A two-sample student *t*-test assuming unequal variance was used to estimate the statistical significance of mean differences.

## Results

We began by generating a transcript sensitive to miRNA-dependent degradation. We generated an artificial miRNA based on the mir6.1 backbone (Chen et al., 2007) designed to target a sequence not found in the *Drosophila* genome, as determined by E-RNAi (Horn & Boutros, 2010) and GESS (Yilmazel et al., 2014) programs, and by nucleotide BLAST search of the guide sequence against the *Drosophila melanogaster* genome (FB2016_04 release). The artificial miRNA was inserted into a 3′ UTR intron of the *mKate2* gene, which let us monitor expression of the miRNA construct. To favor miRNA-directed cleavage rather than end-directed degradation or translational inhibition, three perfectly complementary target sites for the miRNA (Ameres & Zamore, 2013) were incorporated into the 3′ UTR of the Firefly luciferase (*FLuc*) reporter gene. As expected, co-expression of the *FLuc* reporter and the miRNA construct resulted in a large decrease of FLuc protein level as compared with the expression of the *FLuc* reporter construct alone (from 100% to 5.3 ± 0.4%, Fig. 1A).

To determine if we could salvage miRNA-targeted transcripts from degradation, we placed a poly(A) tract of 139 bases immediately 5′ to the miRNA target sites, and downstream of the coding sequence of *FLuc*. We reasoned that following miRNA-guided endonucleolytic cleavage of reporter transcripts the internal poly(A) tract, now located at the newly created 3′ end of the cut transcript, would protect more 5′ sequences from 3′ to 5′ exonucleolytic degradation and allow translation (Wilusz, Wormington & Peltz, 2001). The addition of the internal poly(A) tract into the *FLuc* reporter caused a modest decrease in FLuc expression as compared to the *FLuc* reporter without the poly(A) tract (from 100% to 89.9 ± 6.5%; Fig. 1A). However, when the miRNA and *FLuc* reporter constructs were co-expressed, we found that the *FLuc* reporter with the internal poly(A) tract (i.e., poly(A)+) supported a much higher level of FLuc expression than did the *FLuc* reporter lacking an internal poly(A) tract (i.e., poly(A)−): 81.4 ± 4.0% and 5.3 ± 0.4%, respectively (Fig. 1A).

To test whether reporter transcripts were cleaved by the artificial miRNA we carried out RT-PCR analysis from cells expressing the miRNA and the poly(A)+ or poly(A)− reporter. 1st strand cDNA was reverse transcribed from a position 3′ to the miRNA target sites. A more 5′ region within *FLuc* coding region was then amplified using PCR (Fig. 1B). In such an experiment the amount of PCR product should be proportional to levels of uncut *FLuc* transcript. As illustrated in Fig. 1C, addition of the miRNA caused a 65% decrease in the amount of the *FLuc* RT-PCR product as compared with a no-miRNA control, regardless of whether the transcript did or did not contain an internal poly(A) tract. The presence of the miRNA also did not affect the total levels of polyadenylated mRNAs in *Drosophila* S2 cells as measured by RT-PCR of a non-targeted transcript from the *β glucuronidase* (*βGlu*) gene (Fig. 1C). Together, these results are consistent with a model in which the internal poly(A) tract stabilizes transcripts cleaved at a more 3′ location. With this model in mind we set out to build a transcript in which translation was blocked, but could be liberated by miRNA-dependent cleavage.

Secondary structure in the 5′ -UTR of an mRNA can repress mRNA translation in many contexts (Kozak, 2005; Lammich et al., 2011). We sought to build a secondary structure in the 5′ UTR that could be relieved or prevented from forming by cleavage in the 3′ UTR. To do this, we turned to naturally occurring systems in which a stable RNA structure is generated by interaction between two different RNA molecules. CopT and CopA are well-characterized bacterial RNA molecules. Each forms a hairpin, and they share complementarity over 90 nucleotides. In bacteria, CopA binds *in trans* to CopT, which is located in the leader region of *repA*, forming a structure that prevents translation of *repA* mRNA (Blomberg et al., 1994; Blomberg, Nordstrom & Wagner, 1992). Pairing begins with a transient loop-to-loop interaction (kissing complex), and proceeds to a stable structure, a four-helix junction (Kolb et al., 2000a; Kolb et al., 2000b). This structure blocks ribosome access to ribosomal binding sites in *repA* mRNA (Blomberg et al., 1994). We hypothesized that if CopT and CopA were located in the 5′ and 3′ UTR, respectively, of the same transcript, the formation of a secondary structure resulting from their association would prevent translation of the intervening coding sequence.

To explore this idea we introduced CopT or CopA alone, or in combination, into *FLuc* transcripts. Introduction of a single CopT into the 5′ UTR of *FLuc* mRNA caused a reduction in FLuc expression (from 100% to 65.3 ± 6.7%; Figs. 2A–2B: 2), while placing a single CopA into the 3′ UTR of the transcript had no significant effect on FLuc expression (107.7 ± 26.1%; Figs. 2A–2B: 3). These results are expected, as a strong hairpin structure placed upstream of the start codon, but not in the 3′ UTR, often impedes translational initiation (Kozak, 2005). Importantly, however, a much stronger reduction of FLuc expression was observed when CopT and CopA were inserted into the 5′ and 3′ UTRs of the same *FLuc* transcript, respectively (from 100% to 2.8 ± 0.7%; Figs. 2A–2B: 4). This silencing is likely due to inhibition of translation because transcript levels were similar when one, both or no hairpin structures were present (Fig. 2C).

With these observations in hand we then asked if a miRNA could de-repress or prevent the inhibition of mRNA translation by disrupting or preventing interactions between CopA and CopT. We generated a transcript in which three previously tested target sites for the artificial miRNA (Fig. 1) were placed upstream from CopA in the *FLuc* transcript with both hairpin structures (Fig. 2A: 4). In addition, a poly(A) tract was inserted between the 3′ end of the coding sequence of *FLuc* and the miRNA target sites. This arrangement insures that the cut *FLuc* transcript has a poly(A) tract exposed at the 3′ end. This construct is hereafter referred as the miRNA reporter (Fig. 3A). To assess the efficacy of the miRNA reporter we compared FLuc expression from it with expression from a control transcript in which CopA in the 3′ UTR was replaced with a random sequence of equal length (reporter^CopA−^). The reporter^CopA−^ provides measurements of FLuc expression in the absence of CopA-CopT-mediated repression and thus serves as a reference for reporter performance with and without the miRNA.

We found that the translation of *FLuc* from the miRNA reporter was strongly repressed as compared to the reporter^CopA−^ (from 100% to 4.4 ± 0.5%, Fig. 3B). This repression was dramatically reduced by co-expression of the miRNA (from 4.4 ± 0.5% to 41.9 ± 4.2%). Co-expression of the miRNA with the reporter^CopA−^ had the opposite effect: FLuc translation declined slightly (from 100% to 84.0 ± 3.5%; Fig. 3B). Most importantly, RT-PCR analysis shows that while the presence of the miRNA causes a significant decrease in the levels of uncleaved miRNA reporter transcript as compared with a no miRNA control (from 100% to 43%, Fig. 3C), translation of *FLuc* from the miRNA-expressing cells was increased 10× over the no-miRNA control (Fig. 3B). Together these observations argue that miRNA guided cleavage of the reporter’s transcript at specific target sites reverses or prevents translational repression, thereby resulting in a miRNA cleavage reporter with a positive translational readout.

## Discussion

Several models can explain how translational repression of the reporter is relieved by cleavage. First, mRNA cleavage may facilitate dissociation (CopA would now be linked only non-covalently to the rest of the transcript) or degradation of CopA from the CopA-CopT complex, thereby creating an unstructured 5′ UTR within which scanning-mediated translational activation can occur. Relief from repression could also occur through a kinetic mechanism in which translation only occurs on those transcripts that are cleaved before CopA and CopT interact to form an irreversible block to translation, the four-helix junction (Kolb et al., 2000a; Kolb et al., 2000b). In the first model, cleavage would be expected to promote the translation of both repressed and newly emerged, but not yet repressed transcripts, giving rise to levels of FLuc similar to those seen with the reporter lacking CopA, reporter^CopA−^. However, this was not the case, as illustrated in Fig. 3B. In contrast, in the second model, RNA cleavage can only rescue translation of the fraction of transcripts not already irreversibly locked into a CopA-CopT complex. Indeed, the cut miRNA reporter expressed two times less FLuc than the cut reporter^CopA−^ (41.9 ± 4.2% and 84.0 ± 3.5%; Fig. 3B). Together these results suggest that the miRNA-dependent translational activation of *FLuc* observed in the miRNA reporter is not due to de-repression *sensu stricto*, but rather due to prevention of translational repression in the first place.

To our knowledge, the CopA-CopT reporter is the first example of a one-component positive-readout reporter of site-specific mRNA cleavage. Its design offers several advantages over two-component positive reporters. First, it does not require the constitutive expression of an exogenous repressor protein, which can result in off-target effects and potentially lead to unwanted immune responses *in vivo*. Second, the reporter is expected to respond rapidly because its activation does not depend on degradation of a repressor protein. Third, any reporter protein can be used since CopT and CopA binding will repress translation of any intervening coding sequence. Finally, the specificity of the reporter is in principle adjustable. Inclusion of multiple target sites for a single miRNA creates a reporter of high specificity, while introduction of target sites for different miRNAs may allow for the creation of reporters with broader specificity. Use of complementary sequences other than CopT and CopA may allow the system to be tuned in various ways.

The reporter can in principle monitor presence and activity of any small RNA that guides or directly performs cleavage of an RNA in the cytoplasm. piRNA, rasiRNA, and en-siRNA bind to several Argonaute proteins (PIWI, Aubergine, Ago3 and Ago2) that are capable of endonucleolytic cleavage (Hutvagner & Simard, 2008). The germline-specific expression of piRNAs and the sequence diversity of piRNAs, rasiRNA, and en-siRNAs impede their studies. The details of their biogenesis and function are still under intense investigation (Ameres & Zamore, 2013; Goh et al., 2015; Han et al., 2015; Zhang et al., 2015). A reporter that distinguishes between a site-specific RNA cleavage and general RNA degradation can help to clarify details of piRNA, rasiRNA, and en-siRNA function in the cytoplasm. The reporter can also be used to fine-tune the efficacy of synthetic short hairpin RNA (s-shRNA) (Stevenson et al., 2013). RNA interference is a potent technology to repress disease-causing genes by degrading their mRNAs with s-shRNA (Davidson & McCray, 2011; Kwon et al., 2014; Yoo et al., 2007). Both s-shRNAs and en-siRNAs are loaded into Ago2-RISC and can guide specific endonucleolytic cleavage of RNA molecules. Variants of the described reporter may prove useful in assessing the efficacy of an individual shRNA as well as in optimizing the delivery systems for RNAi-based drugs.

The design of the reporter may also support therapeutic applications. Specific changes in expression patterns of miRNAs have been linked to development of multiple human diseases, including cancers (Hayes, Peruzzi & Lawler, 2014; Lee & Dutta, 2009). Some cancers downregulate the expression of specific miRNAs as compared with normal cells. These cells can be selectively targeted for death following delivery of suicide genes that contain target sites for these miRNAs (Lee, Rennie & Jia, 2009; Wu et al., 2009). In other cancer contexts expression of specific miRNAs known as onco-miRNAs is upregulated, and may play a causal role in carcinogenesis (Medina, Nolde & Slack, 2010). Introduction of small RNAs that are complementary to onco-miRNAs provides a way to block the tumor-promoting functions of these miRNAs (Cheng et al., 2015; Medina, Nolde & Slack, 2010). It will be interesting to see if the positive reporter described here, with an onco-miRNA-dependent suicide gene incorporated between CopT and CopA, can be used to selectively kill onco-miRNA overexpressing cells. While the CopT and CopA system provides a ∼10-fold dynamic range, there are several challenges to implementation of such a system. First, there is significant background expression of the coding region in the absence of the target miRNA. This may cause unwanted killing of normal cells. Second, the onco-miRNA levels must be low enough in non-tumor cells to prevent off-target death, but high enough in tumor cells to cause death. Success in the face of these issues will require detailed knowledge about onco-miRNA expression levels and the ability to accurately deliver defined levels of the suicide transgene to the relevant cell types, but not others.

##  Supplemental Information

10.7717/peerj.3602/supp-1 Supplemental Information 1 Supplementary Tables All raw data from transfection experiments: FLuc/RLuc ratios.

10.7717/peerj.3602/supp-2 Supplemental Information 2 Plasmid sequences Sequence files in GenBank format as they were submitted to GenBank. The records will be available after publication of this manuscript. The plasmid features are mapped in the files: FLuc coding sequence, CopA, CopT, miRNA targets, and etc.

## Additional Information and Declarations

### DNA Deposition

The following information was supplied regarding the deposition of DNA sequences: Sequences are available in GenBank (KY412813, KY412814, and KY412815) and in the Supplemental Information.

### Data Availability

The following information was supplied regarding data availability: The raw data are located in the Supplementary Materials.
