# Supplementary material for: A positive readout single transcript reporter for site-specific mRNA cleavage
Source: PeerJ. 2017 Jul 20;5:e3602. doi: 10.7717/peerj.3602 (PMC5522606; doi:10.7717/peerj.3602)
Supplement: Supplemental Information 1 — All raw data from transfection experiments: FLuc/RLuc ratios. [file peerj-05-3602-s001.pdf]

Supplementary table 1 FLuc/RLuc expression ratios from two kinds of FLuc transcripts: with and without the poly(A) tract inserted upstream of the mRNA cleavage site.

#	Poly(A)	miRNA	FLuc/RLuc
1	–	–	1.00
2	–	–	1.00
3	–	–	1.00
4	–	–	1.00
5	–	–	1.00
1	–	+	0.05
2	–	+	0.05
3	–	+	0.05
4	–	+	0.06
5	–	+	0.05
1	+	–	0.82
2	+	–	0.87
3	+	–	0.96
4	+	–	0.88
5	+	–	0.95
1	+	+	0.85
2	+	+	0.76
3	+	+	0.78
4	+	+	0.82
5	+	+	0.86

Drosophila S2 cells were transfected with two kinds the Firefly Luciferase (FLuc) plasmids: with and without the internal poly(A) tract of 139 nucleotides inserted upstream of the miRNA targets sites (poly(A) – and poly(A) +, respectively: Fig. 1B); and a separate plasmid expressing *Renila* Luciferase (RLuc). An additional plasmid expressing the miRNA was either absent or present in transfected cells (miRNA – and miRNA +, respectively). In five transfections, FLuc/RLuc ratios were quantified for each experimental setting and normalized to that of the FLuc plasmid without the poly(A) track in the absence of miRNA.

Supplementary table 2 FLuc/RLuc expression ratios from FLuc transcripts with four different arrangements of hairpin structures around its coding sequence.

#	5'UTR	Coding sequence	3'UTR	FLuc/RLuc
1		FLuc		1.00
2		FLuc		1.00
3		FLuc		1.00
4		FLuc		1.00
1	CopT	FLuc		0.73
2	CopT	FLuc		0.63
3	CopT	FLuc		0.58
4	CopT	FLuc		0.68
1	–	FLuc	CopA	1.40
2	–	FLuc	CopA	0.86
3	–	FLuc	CopA	1.18
4	–	FLuc	CopA	0.87
1	CopT	FLuc	CopA	0.04
2	CopT	FLuc	CopA	0.03
3	CopT	FLuc	CopA	0.02
4	CopT	FLuc	CopA	0.02

Drosophila S2 cells were transfected with four kinds the Firefly Luciferase (FLuc) plasmids: with no hairpin structure; with CopT in the 5'UTR; with CopA in the 3'UTR; and with both CopT and CopA in the 5'UTR and 3'UTR, respectively (see Fig. 2A). *Renila* Luciferase (RLuc) was expressed from a separate plasmid. In four transfections, FLuc/RLuc ratios were quantified for each experimental setting and normalized to that of the FLuc plasmid without any hairpin structure.

Supplementary table 3 FLuc/RLuc expression ratios from two kinds of FLuc transcripts: non-functional reporter^{CopA} and fully functional reporter of mRNA cleavage.

#	FLuc transcript	miRNA	FLuc/RLuc
1	reporter^{CopA}	–	1.00
2	reporter^{CopA}	–	1.00
3	reporter^{CopA}	–	1.00
4	reporter^{CopA}	–	1.00
5	reporter^{CopA}	–	1.00
1	reporter^{CopA}	+	0.89
2	reporter^{CopA}	+	0.85
3	reporter^{CopA}	+	0.82
4	reporter^{CopA}	+	0.81
5	reporter^{CopA}	+	0.84
1	reporter	–	0.04
2	reporter	–	0.04
3	reporter	–	0.05
4	reporter	–	0.04
5	reporter	–	0.05
1	reporter	+	0.43
2	reporter	+	0.38
3	reporter	+	0.39
4	reporter	+	0.45
5	reporter	+	0.46

Drosophila S2 cells were transfected with two kinds of Firefly Luciferase (FLuc) plasmids: reporter^{CopA}, reporter (see Fig. 3A), and a separate plasmid expressing *Renila* Luciferase (RLuc). An additional plasmid expressing the miRNA was either absent or present in transfected cells (miRNA – and miRNA +, respectively). In five transfections, FLuc/RLuc ratios were quantified for each experimental setting and normalized to that of Reporter^{CopA} in the absence of the miRNA.
